# Fatal *Balamuthia mandrillaris* Encephalitis

**DOI:** 10.1155/2019/9315756

**Published:** 2019-01-31

**Authors:** Binoy Yohannan, Mark Feldman

**Affiliations:** Department of Internal Medicine, Texas Health Presbyterian Hospital Dallas, Dallas 75231, USA

## Abstract

*Balamuthia mandrillaris* is a rare cause of granulomatous meningoencephalitis associated with high mortality. We report a 69-year-old Caucasian female who presented with a 3-day history of worsening confusion and difficulty with speech. On admission, she was disoriented and had expressive dysphasia. Motor examination revealed a right arm pronator drift. Cerebellar examination showed slowing of finger-nose testing on the left. She was HIV-negative, but the absolute CD4 count was low. Neuroimaging showed three cavitary, peripherally enhancing brain lesions, involving the right frontal lobe, the left basal ganglia, and the left cerebellar hemisphere. She underwent right frontal craniotomy with removal of tan, creamy, partially liquefied necrotic material from the brain, consistent with granulomatous amoebic encephalitis on tissue staining. Immunohistochemical studies and PCR tests confirmed infection with *Balamuthia mandrillaris*. She was started on pentamidine, sulfadiazine, azithromycin, fluconazole, flucytosine, and miltefosine. The postoperative course was complicated by an ischemic stroke, and she died a few weeks later.

## 1. Introduction

*Balamuthia mandrillaris* is a free-living heterotrophic amoeba found in soil. This organism can cause a rare and usually fatal granulomatous amebic encephalitis (GAE) in both immunocompetent and immunocompromised patients. Since the time that *Balamuthia mandrillaris* was first isolated, approximately 200 cases had been reported worldwide [1], and in the United States, there have been 94 cases reported to the CDC from 1976 to 2014 (http://www.cdc.gov). We report a case of *Balamuthia* encephalitis in an elderly woman who presented with altered mental status.

## 2. Case Presentation

A 69-year-old Caucasian female was brought to the emergency room by her family for worsening confusion that began 3 days prior to admission. Her son reported that she had not been acting like herself, had been confused, and had been sending him some gibberish text messages. They also noticed that the patient was very quiet, which was unusual for her. She had trouble finding words and her speech did not make sense. She seemed to be indifferent and inattentive at times and had urinary incontinence. There was no history of fever, chills, headache, nausea, vomiting, photophobia, diplopia, or seizure activity. She denied any skin rash or ulcers. She liked to garden in her free time.

Her general physical examination was unremarkable, and she was well-nourished. Vital signs were within normal limits. Ophthalmological examination revealed bilateral 3 mm reactive pupils with full conjugate extraocular movements and no nystagmus or ptosis. Visual acuity was 20/50 in the right eye and 20/100 in the left eye. Funduscopic examination revealed slightly blurred disk margins on the left, with sharp disk margins on the right; the retinal vascularity was normal. She was alert and cooperative and was oriented to self and time but not to place. She could add simple numbers but had difficulty with serial seven subtractions. She was slow to spell “world” backward. She spoke in short phrases and occasionally sentences. Her speech was fluent, but there was intermittent misuse of words and expressive dysphasia. Cranial nerves 2 through 12 were intact, except for a right eyelid droop. Motor examination revealed a slight pronator drift of the right arm. She had 5/5 muscle power in the upper and lower extremities, and her deep tendon reflexes were symmetric. Cerebellar examination showed slowing of the finger-nose testing and rapid alternating movements on the left but without tremor or dysmetria.

**Figure 1:**
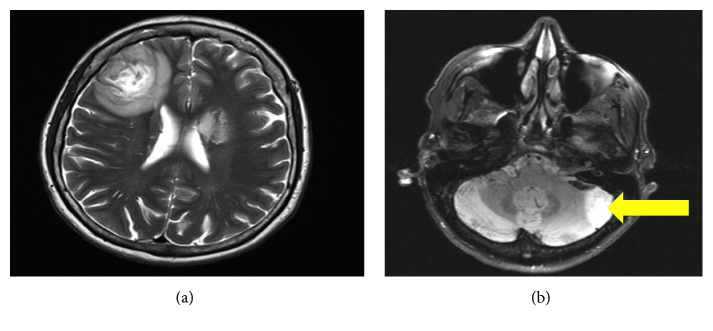
(a) CT head with contrast showing a 3 cm region of heterogeneous low density in the right frontal lobe. A second, 1.5 to 2 cm region of low attenuation in the anterior aspect of the left basal ganglia consistent with an intraaxial mass lesion compressing the left lateral ventricle. (b) MRI brain with contrast showing a central cavitary, contrast-enhancing lesion involving the lateral left cerebellar hemisphere (see arrow).

**Figure 2:**
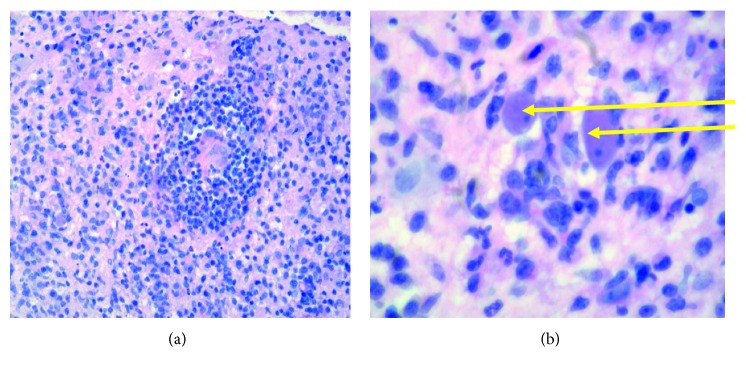
(a) Right frontal lobe histopathology (low power) showing dense lymphocytic infiltrate with granuloma formation. Abundant mononuclear cells are present. AFB and fungal stains were negative. (b) Hematoxylin and eosin stain, with arrows demonstrating amoebic trophozoites under high power magnification (×100).

The complete blood count, metabolic panel, and inflammatory markers (ESR and CRP) were normal. She was HIV-negative, but the absolute CD4 count was 250/mm^3^ (reference range 500–1500/mm^3^). Neuroimaging showed centrally cavitary, peripherally enhancing intra-axial brain lesions, involving the right frontal lobe, the left basal ganglia, and the lateral aspect of the left cerebellar hemisphere (Figure 1). She was started on IV dexamethasone (10 mg bolus) followed by 4 mg every 6 hours for cerebral edema. She underwent right frontal craniotomy the next day with removal of tan, creamy, partially liquefied necrotic material, which was sent for histopathology and cultures. The final histopathology was consistent with granulomatous amebic encephalitis (Figure 2). Bacterial cultures were negative. Immunohistochemical and real-time PCR tests performed at the Centers for Disease Control and Prevention, Atlanta, GA, documented positive results for *Balamuthia mandrillaris* amoeba and negative immunohistochemical and real-time PCR results for *Naegleria fowleri* amoeba and *Acanthamoeba* species amoeba. DNA sequencing data were unavailable. As per the CDC recommendations, she was started empirically on a six-drug regimen of pentamidine (which has amoebastatic activity *in vitro*), sulfadiazine, azithromycin, fluconazole, flucytosine, and miltefosine (which has amebicidal activity *in vitro*). These treatment recommendations were mostly based on a few *Balamuthia* survivor case reports in the literature. Her hospital course was complicated by an ischemic stroke on postoperative day 3, and she clinically deteriorated with worsening mental status. Given the poor prognosis, her family decided to pursue hospice care, and she died a few weeks later.

## 3. Discussion

*Balamuthia mandrillaris* is a rare cause of granulomatous amebic encephalitis (GAE). This free-living amoeba was first isolated from the brain of a mandrill baboon that died at San Diego Zoo from a mysterious neurological illness [2]. *B. mandrillaris* is considered a close relative of *Acanthamoeba* and is placed in the family Acanthamoebidae [3]. *Balamuthia mandrillaris* may also harbor pathogenic bacteria in the natural environment such as *Listeria monocytogenes, Vibrio cholerae*, *E. coli* O157 : H7, *Mycobacterium avium* complex, *Burkholderia pseudomallei*, and *Legionella pneumophila*, thus potentiating their virulence and environmental survival [4]. The worrisome feature about *Balamuthia mandrillaris* is that it can cause GAE irrespective of the status of the host immune system, and this is a matter of grave concern as there are no definitive treatments available [5, 6]. Males are affected 2.5 times more frequently than females, perhaps because they are more exposed to soil through outdoor activities [5].

Those at high risk for this infection include people with HIV/AIDS, cancer, liver disease, or diabetes mellitus, people receiving immunosuppressive drugs, and alcoholics [1, 6]. This patient had idiopathic CD4 lymphopenia, which could have predisposed her to this rare infection. Patients at the extremes of age (under 15 years and over 60 years) appear to be more susceptible, which may be attributed to their weaker immune systems [7]. Most cases of BAE have been reported from the warmer regions, with South America and the southwestern United States (California, Texas, and Arizona) having the highest incidence [5, 8].

*Balamuthia mandrillaris* can be isolated from soil, and contact with contaminated soil is considered a major risk factor for contracting *Balamuthia mandrillaris* amebic encephalitis (BAE) [9, 10]. Our patient was a gardener; hence, the likely port of entry could have been through the skin. The life cycle of *B. mandrillaris* has two stages, the vegetative trophozoites and the dormant cyst. The trophozoites are the infective form, and they enter the human body either through inhalation or through a break in the skin. In most cases, a primary site of infection is not identified, but almost half of the survivors of *B. mandrillaris* GAE reported having an antecedent cutaneous lesion. Hence, it can be postulated that detection of skin lesions can lead to earlier diagnosis and more prompt antimicrobial drug treatment, which potentially could increase the patient's chances of survival [11]. The incubation period varies from 1 to 30 days (with an average of 8.5 days), and the amoeba can invade the central nervous system by hematogenous dissemination causing GAE [5]. Circulating amoebae most likely gain access to the CNS through the blood-brain barrier (BBB). However, if *B. mandrillaris* is isolated from the CSF, a port of entry could be through the highly vascular choroid plexus [12]. The BBB is highly selective, restricting the entry of pathogens, although recent studies have shown that the human brain microvascular endothelial cells produce interleukin-6 (IL-6) in response to *B. mandrillaris* infection, and this may play a role in the traversal of the BBB [13]. Human-to-human disease transmission of the pathogen can occur through organ transplantation, and thus, brain-dead victims of *Balamuthia* encephalitis are not suitable organ donors [14, 15].

Unlike in the patient reported here, BAE often has a slow, insidious onset, which then develops into a subacute or chronic disease over several months to years. However, infections associated with organ transplantation have a rapid clinical course likely related to severe immunosuppression [14, 15]. Patients often present with behavioral and personality changes, confusion, seizures, and cranial nerve dysfunction. As the disease progresses, patients can have symptoms of increased intracranial pressure. Neuroimaging of GAE typically shows multiple, well-defined, focal, ring-enhancing, space-occupying lesions, with perilesional edema; ventriculomegaly and hydrocephalus have also been reported. Another hallmark is hemorrhage into the mass lesion [16, 17]. In patients with GAE due to *B. mandrillaris,* any cortical lobe can be involved: temporal (51%), frontal (41%), occipital (31%), and parietal (21%). Among extracortical sites, the cerebellum, thalamus, basal ganglia including the caudate nucleus, and the brainstem are the most favored sites [18]. As in our patient, angiitis secondary to amebic invasion can cause small vessel occlusions, resulting in cerebral infarction.

Balamuthiasis is difficult to diagnose because of the rarity of this disease, the nonspecific symptoms and signs, and lack of awareness among clinicians. In cases of suspected *B. mandrillaris* infection, the CDC in the USA can be contacted for immediate consultation. Free-living amoebae are rarely isolated from the CSF, and thus, an antemortem diagnosis without brain biopsy is challenging. The CSF analysis when performed often reveals nonspecific lymphocytic pleocytosis with normal/low glucose and elevated protein levels. Microscopic examination of tissue sections from biopsy specimens stained with hematoxylin and eosin (H&E) or periodic acid-Schiff (PAS) may demonstrate amebic trophozoites and/or cysts with morphology typical of *Balamuthia* scattered in the perivascular space [19]. Other hallmarks on biopsy include characteristic granuloma formation with CD4 and CD8 T cells, B lymphocytes with few plasma cells, macrophages, and multinucleated giant cells. The major disadvantage of microscopy is the need for significant expertise regarding the morphological characteristics of *B. mandrillaris* and the availability of a high-quality tissue sample. Immunohistochemistry has been widely used in the detection of the morphological forms of the parasite, and this technique also reliably differentiates *B. mandrillaris* from *Acanthamoeba* spp [20]. PCR-based methods analyze the organism's ribosomal RNA gene sequences (16s rRNA or 18s rRNA), and they are more sensitive and specific in diagnosing *B. mandrillaris*, requiring little pathogen-specific expertise [3]. The major advantage of PCR-based techniques is that the genetic material can be detected either in the CSF or blood, and it eliminates the need for invasive tissue biopsy. A real-time PCR is superior to conventional PCR as it is quicker and reduces the risk of contamination. To avoid false-positive results due to cross reactivity and false-negative results due to novel variants or mutants in the target gene, the identification by PCR should involve the detection of at least 2 independent areas of the pathogen's genome in parallel. A novel target (RNase P gene) of the *B. mandrillaris* primer set can be combined with the 18S rRNA gene in a duplex real-time PCR assay to ensure maximum specificity [21]. A triplex real-time TaqMan PCR assay has been developed by the CDC, which can simultaneously detect *Acanthamoeba* spp., *B. mandrillaris*, and *N. fowleri*. The triplex assay has high specificity and a rapid test completion time of less than 5 hours [22]. Nested PCR can be used to detect the organism directly in soil and water samples [23]. Alternatively, metagenomic deep sequencing is a rapid diagnostic tool for patients with difficult-to-diagnose *Balamuthia* encephalitis [24].

*Balamuthia* GAE has a high case fatality rate of more than 95%, with only 10 reported cases of patients surviving this deadly CNS infection. Various combinations of antimicrobials have been used with disappointing results. The failure of antimicrobial therapy is primarily due to their poor CSF penetration and the thick cell wall of the amebic cyst. Pentamidine, flucytosine, fluconazole, sulfadiazine, and either azithromycin or clarithromycin have been used in patients who have survived the infection [25]; all of these drugs were given to our patient. Miltefosine, previously used for leishmaniasis, has shown some promise and is approved by the U.S. Food and Drug Administration as an investigational treatment for BAE [26]; that too was given to our patient. The optimal duration of combination drug therapy for GAE is unknown, but most survivors had been treated for a few months up to more than 5 years. Survivors of GAE cases often suffer from permanent neurocognitive disorders due to the extensive cerebral edema that develops during the course of the illness. In the absence of effective treatment, prevention is the most effective strategy for BAE. As this infection is more common in agricultural workers or persons with regular contact with soil, certain simple precautionary measures should be taken to prevent *B. mandrillaris* from entering the host. For example, individuals with skin lesions should wear protective clothing while working in soil.

Three other species of free-living amoeba, *Acanthamoeba* spp., *Sappinia pedata*, and *Naegleria fowleri*, have also been implicated in central nervous system infections. *Acanthamoeba* spp. and *Sappinia pedata* can cause granulomatous amebic encephalitis (GAE), similar to BAE, particularly in immunocompromised hosts [27, 28]. In acanthamoebiasis, the clinical outcome is impacted by surgical debulking, early initiation of antimicrobial therapy, and the immune status of the host. Voriconazole and miltefosine are two promising oral agents with *in vitro* activity against *Acanthamoeba*, and they have excellent brain parenchymal and CSF penetration. *Naegleria fowleri*, commonly referred to as “brain-eating amoeba”, causes an acute and fulminant, necrotizing infection of the brain called primary amebic meningoencephalitis (PAM) in children and adults with a history of recent exposure to warm fresh water [29]. There is a high mortality rate probably exceeding 98% patients in patients with PAM. Early triple therapy with intravenous amphotericin B and fluconazole and oral administration of rifampicin can offer some hope of cure for this devastating disease [30].

## 4. Conclusions

*Balamuthia mandrillaris* is a free-living amoeba found in soil that can cause fatal granulomatous amebic encephalitis in both healthy and immunocompromised patients. Although *Balamuthia* amebic encephalitis is rare, clinicians should maintain a high index of clinical suspicion of *Balamuthia* in a patient with subacute granulomatous meningoencephalitis and a negative workup for viral, bacterial, and fungal infections, particularly if they had been exposed to soil during work or recreational activities. Brain biopsy is crucial for arriving at the correct diagnosis. Even though multiple combination antimicrobials have been tried, the prognosis of granulomatous amebic encephalitis is dismal. Future studies are essential to identify novel drugs that can penetrate the BBB and treat BAE more effectively.
